# Plasmid Display for Stabilization of Enzymes Inside the Cell to Improve Whole-Cell Biotransformation Efficiency

**DOI:** 10.3389/fbioe.2019.00444

**Published:** 2020-01-10

**Authors:** Yunjeong Park, Jonghyeok Shin, Jinkyeong Yang, Hooyeon Kim, Younghun Jung, Hyunseok Oh, Yongjoon Kim, Jaehyeon Hwang, Myeongseo Park, Choongjin Ban, Ki Jun Jeong, Sun-Ki Kim, Dae-Hyuk Kweon

**Affiliations:** ^1^Department of Integrative Biotechnology, College of Biotechnology and Bioengineering, Sungkyunkwan University, Suwon-si, South Korea; ^2^Institute of Biomolecule Control, Sungkyunkwan University, Suwon-si, South Korea; ^3^Department of Chemical and Biomolecular Engineering, KAIST, Daejeon, South Korea; ^4^Department of Food Science and Technology, Chung-Ang University, Anseong, South Korea; ^5^Institute of Biologics, Sungkyunkwan University, Suwon-si, South Korea

**Keywords:** Oct-1, DNA binding protein, intracellularly immobilized enzyme system, *Escherichia coli*, whole-cell biotransformation

## Abstract

Recombinant whole-cell biocatalysts are widely used for biotransformation of valuable products. However, some key enzymes involved in biotransformation processes are unstable and cannot be easily expressed in the functional form. In this study, we describe a versatile platform for enzyme stabilization inside the cell: Intracellularly Immobilized Enzyme System (IIES). A 1,2-fucosyltransferase from *Pedobactor saltans* (PsFL) and a 1,3-fucosyltransferase from *Helicobacter pylori* (HpFL), chosen as model proteins, were fused with Oct-1 DNA-binding domain, which mediated the formation of a plasmid–protein complex. Oct-1 fusion enabled both soluble and stable expression of recombinant proteins in the cytoplasm because the fusion proteins were stabilized on the plasmid like immobilized enzymes bound to solid surface. As a result, Oct-1-fusion proteins exhibited significantly greater product titer and yield than non-fusion proteins. Use of fusion proteins PsFL-Oct-1 with C-terminal Oct-1 and Oct-1-PsFL with N-terminal Oct-1 resulted in ~3- and ~2-fold higher 2′-fucosyllactose titers, respectively, than with the use of PsFL alone. When Oct-1 was fused to HpFL, which requires dimerization through heptad repeats, almost two times more 3-fucosyllactose was produced. Fucosyllactose has been used as a food additive because it has various beneficial effects on human health. We anticipate that IIES using Oct-1 fusion protein developed in this study can be applied to stabilize other unstable enzymes.

## Introduction

*Escherichia coli* is generally the first choice of host for the production of proteins and chemicals, because of its fast growth rate, a well-established expression system, high production yield, and low manufacturing cost. In *E. coli* cytoplasm, however, recombinant proteins tend to interact with non-specific hydrophobic patches, leading to the formation of inclusion bodies (Baneyx and Mujacic, 2004). To enhance soluble expression of recombinant proteins, various strategies have been suggested: N-terminus and/or C-terminus truncation, co-expression of chaperones, and fusion protein technologies (Sorensen and Mortensen, 2005). To date, *Schistosoma japonicum* glutathione S-transferase (GST), *E. coli* maltose-binding protein (MBP), *E. coli* N-utilization substance A (NusA), and *E. coli* thioredoxin are commonly used as fusion partners to prevent inclusion body formation (Esposito and Chatterjee, 2006). Although fusion partners provide a general protective effect against insoluble aggregation, some soluble fusions lack detectable activities (Sachdev and Chirgwin, 1999). These soluble aggregates are formed by agglomeration of misfolded proteins of interest, while precipitation is prevented by the presence of the soluble fusion partners (Nomine et al., 2001). Thus, an alternative fusion system should be developed to increase soluble expression levels and simultaneously block agglomeration formation between misfolded target proteins.

As model proteins, we chose two fucosyltransferases (FTs). FTs transfer the fucosyl residue of GDP-l-fucose to lactose, leading to the formation of fucosylated human milk oligosaccharides (HMOs). Microbial production of HMOs has received great attention, as they show beneficial effects on human health, such as prevention of pathogenic infection, modulation of the immune system, and prebiotic effects (Bode, 2015). Among various HMOs present, almost half are fucosylated, and the most abundantly present fucosylated HMOs are 2′-fucosyllactose (Fuc-α1,2-Gal-β1,4-Glc; 2′-FL) and 3-fucosyllactose (Gal-β1,4-(Fuc-α1,3-)Glc; 3-FL) (Thurl et al., 2010). Although all 1,2-FTs are monomers and their activities are related to intrinsic solubility, all 1,3-FTs contain various lengths of heptad repeats mediating dimerization, which is crucial for substrate binding (Sun et al., 2007). Thus, 1,2-, and 1,3-selective FTs were included as the model proteins, not only because the final products are important but also because their structural architectures are very distinct from each other. However, the reactions catalyzed by FTs have been recognized as limiting steps because these enzymes are hardly expressed in a functional form and are rather unstable under process conditions (Chin et al., 2015; Choi et al., 2016; Yu et al., 2018).

Enzyme immobilization provides an effective way to circumvent the concerns related to aggregation, by improving enzyme stability against temperature, solvents, pH, and impurities (Sheldon, 2007; Xie et al., 2009). Also, enzyme immobilization allows reutilization of biocatalyst and hence reduces the cost of biocatalyst production. Although the *in vitro* technology of enzyme immobilization has been intensively studied, protein engineering efforts to immobilize enzymes *in vivo* have been few (Steinmann et al., 2010). Therefore, we sought to develop a system in which the target enzyme is synthesized by *E. coli* and simultaneously immobilized to the surface of a stable molecule that is produced by *E. coli*. Because DNA is a very stable molecule in *E. coli* cytoplasm, the enzyme of interest can be potentially stabilized if the enzyme is attached to plasmids. Recently, a plasmid display system using human Oct-1 DNA-binding domain (DBD) was successfully developed (Park et al., 2013). Human Oct-1 is a transcription factor involved in the regulation of various housekeeping genes (Segil et al., 1991). Oct-1 DBD consists of an N-terminal POU-specific domain, a short flexible linker, and a C-terminal POU homeodomain. Although each domain does not have high binding affinity of each domain to target DNA binding sequences (BS, 5′-ATGCAAAT-3′) is not high enough, Oct-1 DBD can bind to BS with high affinity (K_D_ = 9 × 10^−11^ M) via cooperative binding of both domains (Klemm and Pabo, 1996). Moreover, Oct-1 DBD itself exhibited both high expression level and high solubility (Park et al., 2013). These results suggest that Oct-1 DBD is a good fusion tag to demonstrate intracellularly immobilized enzyme system (IIES) among other DBDs with their relatively large size, or complex structures that are difficult to produce in the cytoplasm of *E. coli* (Yesudhas et al., 2017). Furthermore, we found that Oct-1 DBD binds non-specifically to DNA without BS, enabling to overcome potential stoichiometric mismatch between the number of BS and the number of expressed fusion proteins. In this study, a novel whole-cell biotransformation process with IIES was developed to enhance production yield of 2′-FL and 3-FL, whose production was catalyzed by *Pedobactor saltans* 1,2-FT (PsFL) and *Helicobacter pylori* 1,3-FT (HpFL), respectively.

## Materials and Methods

### Strains and Plasmids

All the strains and plasmids used in this study are listed in Table 1. All cloning experiments were performed using a ligation-independent cloning method with T4 DNA polymerase (New England Biolabs). The PCR amplification with Q5 High-Fidelity DNA Polymerase (New England Biolabs) was set as initial denaturation step at 98°C for 30 s, followed by 35 repetitions of the following cycle: denaturation step, 98°C, 10 s; annealing step, 50~55°C, 30 s; and elongation step, 72°C, 30 s/kb. The final step took place at 72°C for 2 min. Plasmids pOct-1-PsFL, pPsFL-Oct-1, pBSx-Oct-1-PsFL, and pBSx-PsFL-Oct-1 were constructed as shown in Supplementary Figures 1, 2. Plasmid pOct-1-PsFL was constructed in five cloning steps. First, a DNA fragment coding for Oct-1 DBD was amplified with primers pCOLA-Oct-1 I FW and pCOLA-Oct-1 I BW using human cDNA as the template. This DNA fragment combined with a linearized plasmid pCOLADuet-1 amplified with primers pCOLA-Oct-1 V FW and pCOLA-Oct-1 V BW to construct pCOLA-Oct-1. Second, Plasmid pBS3-Oct-1 is identical to pCOLA-Oct-1 except that it contains 3 repeats of the Oct-1-specific DNA binding sequence (3 OBS). To make this change, a DNA fragment containing pCOLA-Oct-1 was amplified with primers pCOLA-BS FW and pCOLA-BS BW to be combined by T4 DNA polymerase. Third, a DNA fragment coding for PsFL was amplified with primers pCOLA-Oct-1PsFL I FW and pCOLA-Oct-1PsFL I BW using pCOLA-PsFL (AP Technology, Suwon, Republic of Korea) as the template. A DNA fragment containing Oct-1 DBD and 3 OBS was amplified with primers pCOLA-Oct-1PsFL V FW and pCOLA-Oct-1PsFL V BW using pBS3-Oct-1 as the template. These two linear DNA fragments were combined using T4 DNA polymerase to construct pOct-1-PsFL intermediate. In a fourth step, pCOLADuet-1 was amplified with primers pCOLA-BS FW and pCOLA-BS BW to construct pCOLA-BS3 by introducing 3 OBS. In the last step, the expression cassette coding for Oct-1-PsFL located in multiple cloning site (MSC) of the first *T7* promoter region (P_T7−1_) was moved to MSC of the second *T7* promoter region (P_T7−2_). To make this change, the expression cassette coding for Oct-1-PsFL was amplified with primers PsFL-Oct-1 I FW and PsFL-Oct-1 I BW. A DNA fragment containing 3 OBS was amplified with primers pCOLADuet V FW and pCOLADuet V BW using pCOLA-BS3 as the template. These two linear DNA fragments were combined using T4 DNA polymerase to construct pOct-1-PsFL. The other three plasmids, pPsFL-Oct-1, pBSx-Oct-1-PsFL, and pBSx-PsFL-Oct-1 were constructed by following the similar procedure (Supplementary Figures 1, 2).

**Table 1:** *E. coli* strains and plasmids used in this study.

**Name**	**Description**	**Reference**
***E. coli***		
TOP10	F^−^*mcrA* Δ(*mrr-hsdRMS-mcrBC*) Φ80*lacZ*ΔM15 Δ*lacX74 recA1 ara*D139 Δ(*ara-leu*)7697 *galU galK rpsL* (Str^R^) *endA1 nupG*	Invitrogen
BL21(DE3)	F^−^ ompT lon *hsdS_*B*_*(${\text{r}}_{\text{B}}^{-}$, ${\text{m}}_{\text{B}}^{-}$) *gal dcm*	New England Biolabs
ΔL M15	BL21star (DE3) Δ*lacZYA Tn7::lacZΔM15YA*	Chin et al., 2015
**Plasmids**		
pCOLADuet-1	Two T7 promoters, ColA replicon, Kan^R^	Novagen
pBCGW	pETDuet-1 + *manC*-*manB* (*Nco*I/*Sac*I) + *gmd*-*wcaG* (*Nde*I/*Kpn*I)	Lee et al., 2009
pCOLA-PsFL	pCOLADuet-1 + WT 1,2-FT from *P. Saltans*	AP Technology (Suwon, Republic of Korea)
pCOLA-HpFL	pCOLADuet-1 + WT 1,3-FT from *H. Pylori*	Yu et al., 2018
pCOLA-Oct-1	pCOLADuet-1 + Oct-1 from human	In this study
pBS3-Oct-1	pCOLA-Oct-1 + 3 repeats of the Oct-1 specific DNA binding sequence	In this study
pOct-1-PsFL intermediate	Intermediate plasmid containing Oct-1-PsFL expression cassette	In this study
pPsFL-Oct-1 intermediate	Intermediate plasmid containing PsFL-Oct-1 expression cassette	In this study
pCOLA-BS3	pCOLADuet-1 + 3 repeats of the Oct-1 specific DNA binding sequence	In this study
pOct-1-PsFL	pCOLA-BS3 + Oct-1-PsFL	In this study
pPsFL-Oct-1	pCOLA-BS3 + PsFL-Oct-1	In this study
pBSx-Oct-1-PsFL	pCOLADuet-1 + Oct-1-PsFL	In this study
pBSx-PsFL-Oct-1	pCOLADuet-1 + PsFL-Oct-1	In this study
pOct-1-HpFL	pCOLA-BS3 + Oct-1-HpFL	In this study
pHpFL-Oct-1	pCOLA-BS3 + HpFL-Oct-1	In this study

Next, plasmid pOct-1-HpFL was constructed by replacing PsFL of pOct-1-PsFL with HpFL. A DNA fragment containing Oct-1 and 3 OBS was amplified with primers pCOLADuet-Oct-1-POI V FW and pCOLADuet-Oct-1-POI V BW using pOct-1-PsFL as the template. A DNA fragment coding for HpFL was amplified with primers Oct-1-HpFL I FW and Oct-1-HpFL I BW using pCOLA-HpFL (Yu et al., 2018) as the template. These two linear DNA fragments were combined using T4 DNA polymerase to construct pOct-1-HpFL. Plasmid pHpFL-Oct-1 was constructed by replacing PsFL of pPsFL-Oct-1 with HpFL, as described above. The primer sets used for amplification of the two DNA fragments are as follows: pCOLADuet-POI-Oct-1 V FW and pCOLADuet-POI-Oct-1 V BW for pPsFL-Oct-1; HpFL-Oct-1 I FW and HpFL-Oct-1 I BW for HpFL. Primers used for plasmid constructions are listed in Supplementary Table 1.

### Analysis of Expression Patterns and Stability of Engineered Proteins

Plasmid pCOLADuet-1, harboring each PsFL or HpFL expression cassette was transformed into *E. coli* BL21(DE3) cell. *E. coli* BL21(DE3) cells were grown at 37°C and 200 rpm in a 250-mL baffled flask containing 50 mL of Lysogeny broth (LB) medium (1% tryptone, 0.5% yeast extract, and 1% sodium chloride) and 50 μg/mL of kanamycin. At the mid-exponential growth phase [optical density (OD_600_) ≈ 0.4–0.6], isopropyl-β-D-1-thiogalactopyranoside (IPTG) was added at a final concentration of 0.1 mM. After IPTG induction, the culture temperature was changed to 16 or 25°C because we wanted to investigate effects of a plasmid display system on soluble expression at both temperatures. After overnight cultivation, *E. coli* cells were harvested by centrifugation at 9,300 g for 10 min, resuspended in Bugbuster protein extraction reagent (Novagen), and lysed by orbital shaking for 30 min at 25°C to collect total protein fraction. Samples were centrifuged at 9,300 g for 10 min to separate soluble fraction from the debris (insoluble fraction). The expression level of the target enzyme was analyzed by 12% sodium dodecyl sulfate-polyacrylamide gel electrophoresis (SDS-PAGE). The quantification of bands intensity was carried out using the densitometry software (TotalLab 1.01, Non-linear Dynamics Ltd., Newcastle upon, UK).

To evaluate thermal stability of PsFL, *E. coli* cells expressing WT PsFL and Oct-1-PsFL were cultivated as described above until OD_600_ value reached 0.4–0.6. After 4 h induction at 25°C with 0.1 mM IPTG, *E. coli* cells were harvested by centrifugation at 9,300 g for 10 to remove IPTG. After IPTG washing, the procedure of centrifugation of culture broth followed by removal of the supernatant containing IPTG, *E. coli* cells were inoculated into fresh LB medium containing 50 μg/mL of kanamycin, and soluble and insoluble fractions were prepared and analyzed as described above.

### *In vitro* Selection of the Plasmid-Protein Complex

Most experimental processes were performed at 25°C to maintain maximum binding affinity of Oct-1 DBD (Lundback et al., 2000) except for centrifugations which were carried out at 4°C. *E. coli* cells expressing Oct-1 DBD fusion proteins were cultivated as described above. After cell cultivation, cells were harvested by centrifugation at 9,300 g for 10 min. The pellets were resuspended in 5 mL Bugbuster protein extraction reagent (Novagen), and then the resuspended cells were agitated at 25°C for 30 min. The resulting cell lysates were centrifuged at 9,300 g for 10 min at 4°C to collect the soluble fraction. The supernatants (soluble fraction) were mixed with Ni Sepharose Fast Flow (GE Healthcare), which was pre-equilibrated with an equilibrium buffer composed of 0.05 M Tris-HCl and 0.3 M NaCl (pH 8.0). After agitation for 30 min at 25°C, the Ni-NTA resin was washed 5 times with 1 column volume of washing buffer composed of 0.05 M Tris-HCl, 0.3 M NaCl, 10 mM imidazole, and 10% glycerol (pH 8.0). The elution was carried out using 500 μL of elution buffer composed of 0.05 M Tris-HCl, 0.3 M NaCl, and 250 mM imidazole (pH 8.0). From the eluted fractions, protein samples were analyzed by SDS-PAGE on a 12% polyacrylamide (w/v) gel followed by western blotting. Bands of His_6_-fused Oct-1 on the polyvinylidene difluoride (PVDF) membranes were probed with anti-polyhistidine-peroxidase antibody (Sigma-Aldrich, A7058). PCR amplification was performed with primer check FW and check BW (Supplementary Table 1) to confirm the presence of correct plasmids. Taq polymerase (Sigma-Aldrich) was used for PCRs to confirm the presence of the plasmid according to the manufacturer's instruction. The PCR amplification was set as initial denaturation step at 94°C for 1 min, followed by 35 repetitions of the following cycle: denaturation step, 94°C, 30 s; annealing step, 53~58°C, 30 s; and elongation step, 72°C, 1 min/kb. The final step took place at 72°C for 2 min. PCR amplification with primers (check FW and check BW) specific to both pBSx-Oct-1-PsFL and pOct-1-PsFL was used to confirm the presence of the plasmid. Primers used for the check PCR are listed in Supplementary Table 1. The PCR amplified DNA products were analyzed by 1% agarose gel electrophoresis with TAE buffer consisting of 40 mM Tris base, 20 mM acetic acid, and 1 mM ethylenediaminetetraacetic acid (EDTA).

### Electrophoretic Mobility Shift Assay (EMSA)

To confirm binding specificity of Oct-1 DBD, the plasmid harboring Oct-1 fused PsFL (Oct-1-PsFL) was transformed into *E. coli* BL21(DE3) competent cell. The Oct-1-PsFL fusion protein was expressed, and His_6_-tag purified as described above. Plasmids pCOLADuet-1, pBSx-Oct-1-PsFL, and pOct-1-PsFL were amplified using primers Gel shift FW and Gel shift BW (Supplementary Table 1). The resulting linearized DNA fragments (~100 ng) were mixed with His_6_-tag purified Oct-1-PsFL fusion protein (~6 μg) before agarose gel electrophoresis, which was carried out as described above.

### Fermentation Conditions

Batch fermentations for the production of 2′-FL and 3-FL were performed in a 250-mL baffled flask containing 50 mL of Riesenberg medium [13.5 g/L KH_2_PO_4_, 4.0 g/L (NH_4_)_2_HpFLO_4_, 1.7 g/L citric acid, 1.4 g/L MgSO_4_·7H_2_O, 10 mL/L trace element solution (10 g/L Fe(III) citrate, 2.25 g/L ZnSO_4_·7H_2_O, 1.0 g/L CuSO_4_·5H_2_O, 0.35 g/L MnSO_4_·H_2_O, 0.23 g/L Na_2_B_4_O_7_·10H_2_O, 0.11 g/L (NH_4_)_6_Mo_7_O_24_, 2.0 g/L CaCl_2_·2H_2_O), pH 6.8] with 20 g/L glycerol and appropriate antibiotics at 37 °C and 200 rpm, according to a previous study (Chin et al., 2015). The ΔL M15, BL21star (DE3) Δ*lacZYA Tn7::lacZ*Δ*M15YA* (Chin et al., 2015), was used as a host for the production of 2′-FL and 3-FL. At the mid-exponential growth phase, IPTG and lactose were added at final concentrations of 0.1 mM and 5 g/L, respectively. After IPTG induction, temperature was changed to 25°C.

### Analytical Methods

Cell growth was measured at OD_600_ using a spectrophotometer (BioMate 3S, Thermo Scientific). Lactose, glycerol, 2′-FL, and 3-FL concentrations were determined by a high-performance liquid chromatography (Waters Corporation) equipped with a Rezex ROA-Organic Acid H+ column (Phenomenex, Torrance, CA) for reversed-phase chromatography. Metabolites were separated at isocratic temperature (50°C) and a flow rate of 0.6 mL/min in 0.01 N H_2_SO_4_, and then passed through a refractive index detector.

## Results

### Effects of Oct-1 Dbd Fusion on Soluble and Stable Expression of 1,2-FT From *P. saltans* (PsFL)

**Figure 1:**
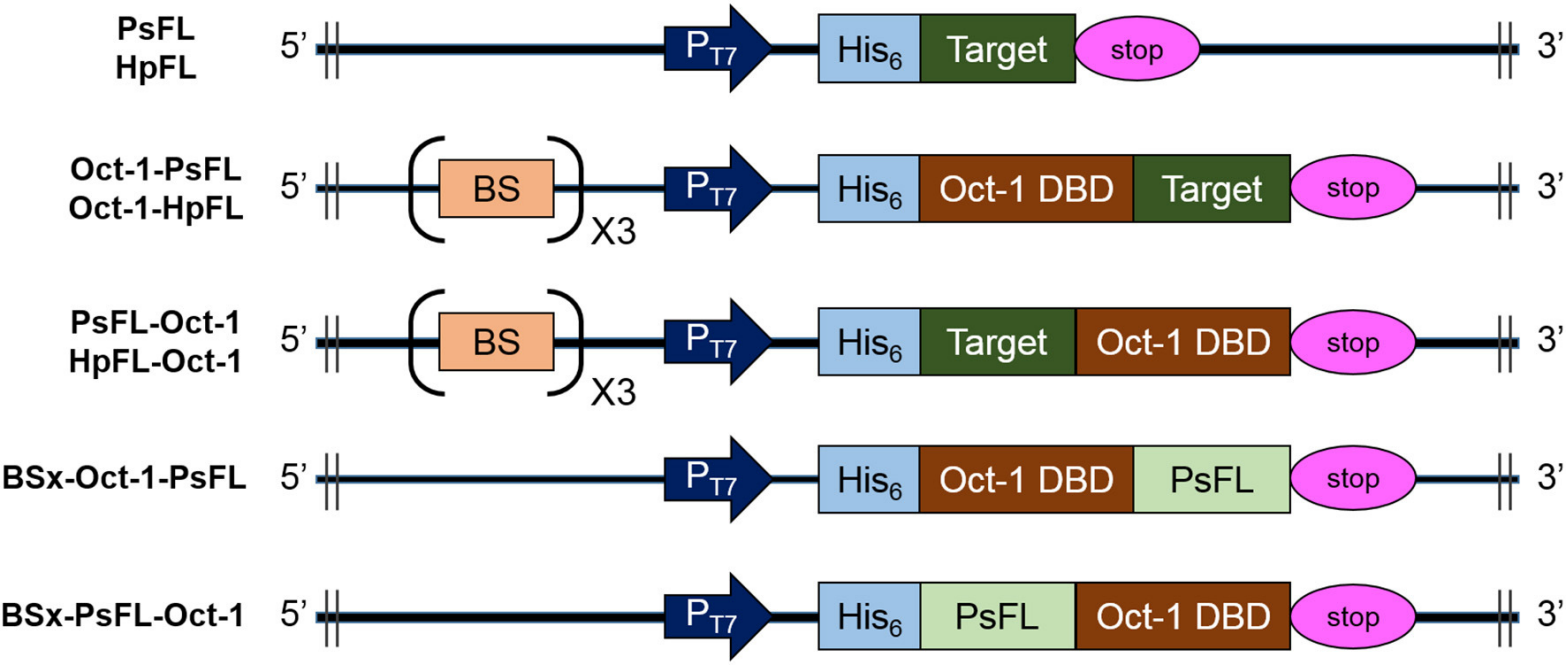
Schematic diagrams of the structures of recombinant PsFL and HpFL expression cassettes. Symbols: *T7* promoter (P_T7_), His_6_-tag (His_6_), translational stop codon (stop), the octameric binding site that was repeated 3 times (BS), Oct-1 DNA-binding domain (Oct-1 DBD), and gene coding for PsFL or HpFL (target).

**Figure 2:**
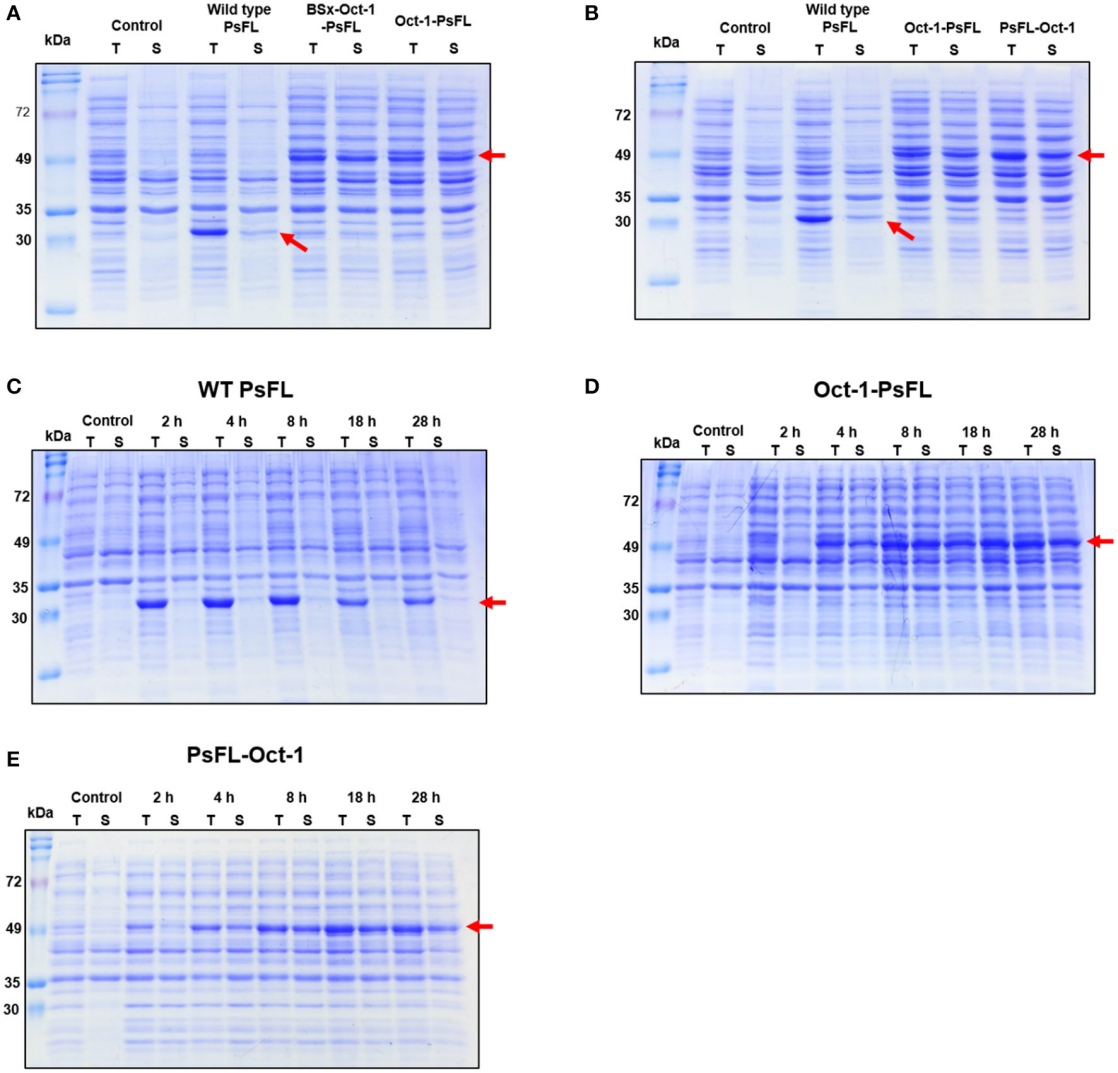
SDS-PAGE analysis of recombinant PsFLs fused with Oct-1 DBD. **(A,B)** Effects of the presence of 3 OBS **(A)** and location of Oct-1 DBD tag **(B)** on soluble expression of recombinant PsFL. *E. coli* BL21(DE3) strains expressing recombinant PsFLs were grown at 16°C for 18 h after 0.1 mM IPTG induction. **(C–E)** Effects of Oct-1 DBD fusion on expression level and patterns of recombinant PsFLs at 25°C. Cell lysates were prepared from *E. coli* BL21(DE3) strains expressing WT PsFL **(C)**, Oct-1-PsFL **(D)**, and PsFL-Oct-1 **(E)** and fractionated into total (T) and soluble (S) fractions. The cell lysate after IPTG induction and supernatant after centrifugation of the cell lysate were named as the total and soluble protein fractions, respectively. The arrow points the protein band of recombinant PsFLs.

To investigate the effects of location of Oct-1 DBD and the presence of 3 repeats of the Oct-1 specific DNA binding sequence (3 OBS), various expression systems were designed and their schematic diagrams were shown in Figure 1. The expression system without any tag was used as a control. The recombinant *E. coli* BL21(DE3) containing the various recombinant PsFL genes was grown at 16°C after IPTG induction, and the total and soluble protein fractions were analyzed by SDS-PAGE. The cell lysate after IPTG induction and supernatant after centrifugation of the cell lysate were named as the total and soluble protein fractions, respectively. Protein bands consistent with a molecular weight of the wild type (WT) PsFL (31 kDa) and recombinant PsFLs (50 kDa) were detected (Figures 2A,B). While most WT PsFLs were not expressed as soluble forms, soluble expression of recombinant PsFLs was significantly improved regardless of the location of Oct-1 DBD and the presence of 3 OBS (Figures 2A,B). Densitometric analysis of PsFL bands showed that 76–89% of PsFL was expressed solubly when Oct-1 DBD was attached whereas only 30% of the WT PsFL was expressed as soluble form (Supplementary Figures 3A,B).

We next examined the effects of Oct-1 DBD fusion on thermal stability of PsFL at 25°C. The induction temperature was increased to 25°C because low induction temperature is not desirable in a large scale fermentation. In addition, we wanted to investigate effects of plasmid display on stability of PsFL at the temperature where difference of stability between WT and recombinant PsFLs became more apparent. When PsFL without any tag was expressed inside the cell and incubated at 25°C for 28 h after induction, the band intensities of the WT PsFL in both total and soluble fractions were decreased continuously over time (Figure 2C and Supplementary Figure 3C). However, when Oct-1 DBD was attached to the N-terminus of recombinant PsFL (Oct-1-PsFL), the PsFL remained soluble and did not show any decrease in band intensity for 28 h after protein expression during initial 8 h (Figure 2D and Supplementary Figure 3D). Attachment of Oct-1 DBD at the C-terminus of PsFL (PsFL-Oct-1) also led to an increase in soluble expression of recombinant PsFL, but to a lesser extent than in the case of N-terminal attachment (Figure 2E and Supplementary Figure 3E). The beneficial effects of Oct-1 DBD fusion on thermal stability of PsFL was further evaluated by comparing expression level of WT PsFL and Oct-1-PsFL after ITPG washing. While soluble expression of WT PsFL was hardly detected after IPTG washing followed by 4 h cultivation, Oct-1-PsFL remained soluble for 6 h (Supplementary Figure 4). These results indicate that IIES via plasmid display allowed proper folding and improved thermostability of the PsFL.

### Binding Ability of Oct-1-PsFL to pCOLADuet-1-Based Plasmids

**Figure 3:**
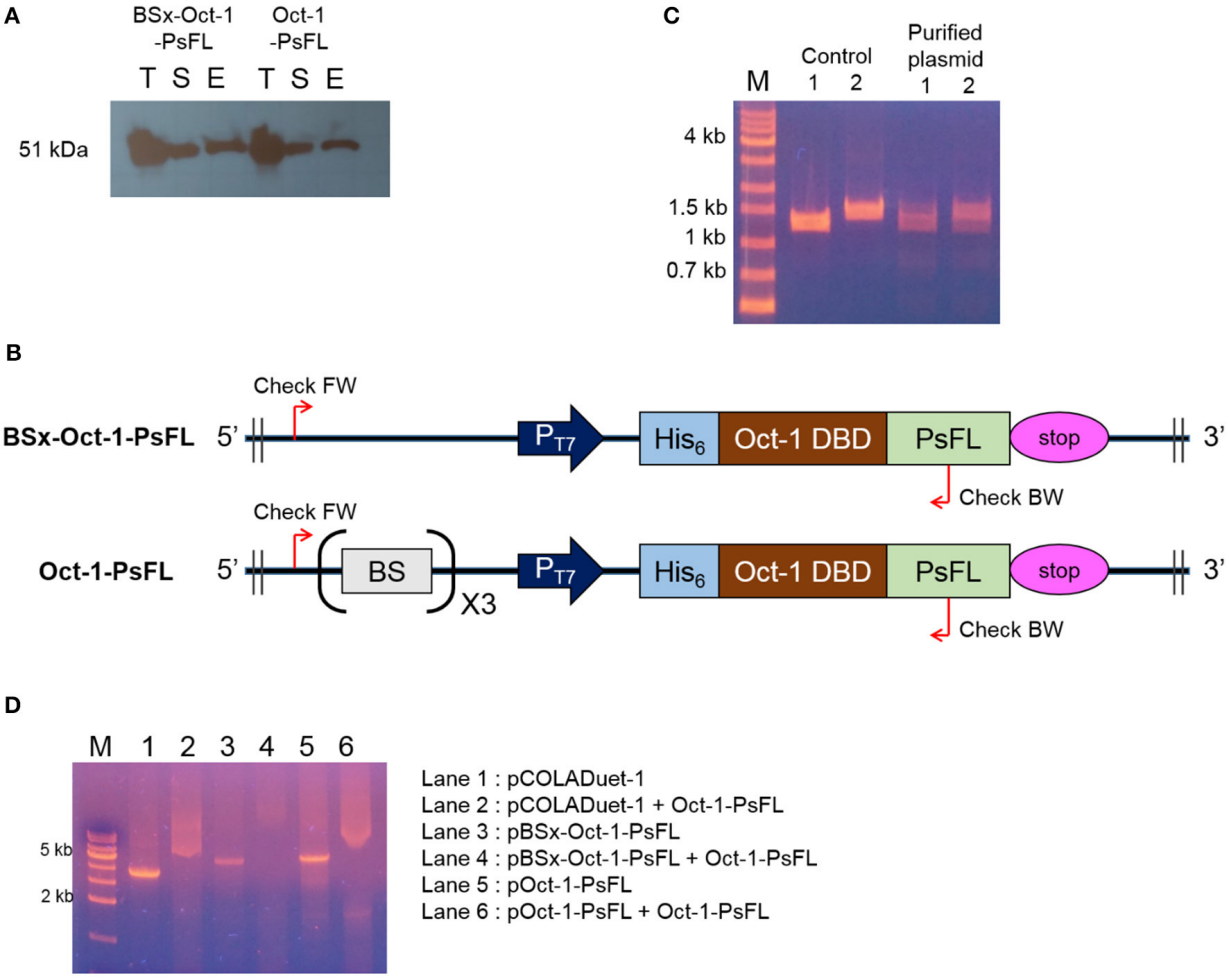
DNA-protein complex formation between PsFL fused with Oct-1 DBD and plasmid. **(A)** Western blotting for Hs_6_-tagged Oct-1-PsFL purified by Ni-NTA chromatography from the cell cultures of *E. coli* BL21(DE3) pBSx-Oct1-PsFL and *E. coli* BL21(DE3) pOct-1-PsFL. Lanes T, S, and E represent the total, soluble, and elution fractions, respectively. **(B)** A depiction of primer binding sites on pBSx-Oct-1-PsFL and pOct-1-PsFL. **(C)** Agarose gel electrophoresis of PCR product of check PCR. PCR products were amplified using primers check FW and check BW annealing to pBSx-Oct-1-PsFL, 1.3 kb (lane 1) and pOct-1-PsFL, 1.5 kb (lane 2). The PCR products were analyzed by 1% agarose gel electrophoresis with the TAE buffer. Plasmids purified directly from *E. coli* TOP10 were used as control. One kilobyte DNA ladder (lane M). **(D)** Electrophoretic mobility shift assay of protein Oct-1-PsFL to plasmids pCOLADuet-1, pBSx-Oct-1-1, and pOct-1-PsFL. (1) pCOLADuet-1 without Oct-1-PsFL; (2) pCOLADuet-1 with Oct-1-PsFL; (3) pBSx-Oct-1-PsFL without Oct-1-PsFL; (4) pBSx-Oct-1-PsFL with Oct-1-PsFL; (5) pOct-1-PsFL without Oct-1-PsFL; (6) pOct-1-PsFL with Oct-1-PsFL. The PCR products were analyzed by 1% agarose gel electrophoresis with the TAE buffer. Binding of proteins to the linearized plasmids resulted in band smearing, whereas clear DNA bands were observed in the absence of DNA-binding proteins.

Interestingly, soluble expression levels of Oct-1-PsFL were almost identical regardless of the presence of 3 OBS in plasmid pCOLADuet-1 (Figure 2A). Therefore, to investigate effects of the presence of 3 OBS on the binding ability of Oct-1 DBD, we performed *in vitro* selection using His_6_-tag fused Oct-1-PsFL protein along with either plasmid pBSx-Oct-1-PsFL without 3 OBS, or pOct-1-PsFL containing 3 OBS. After cell cultivation and disruption, each His_6_-tag fused Oct-1-PsFL protein was purified by Ni-NTA affinity column chromatography. Western blot analysis clearly indicated that the Oct-1-PsFL was selectively purified using affinity resins with high recovery yields (Figure 3A). Then, possible DNA binding to the purified plasmids was assessed using PCR. After recovering plasmids bound to the PsFL-Oct-1 using a plasmid mini-preparation kit, the presence of plasmids was confirmed by check PCR with primers specific to both pBSx-Oct-1-PsFL and pOct-1-PsFL (Figure 3B). It was confirmed that both the plasmids were recovered in the final elute, suggesting that Oct-1-PsFL can bind to pCOLADuet-1-based plasmids, regardless of the presence of 3 OBS (Figure 3C). The ability of PsFL-Oct-1 to bind to plasmids pBSx-Oct-1-PsFL, pOct-1-PsFL, and even pCOLADuet-1 was verified by electrophoretic mobility shift assay (Figure 3D). We note that 3 OBS-independent binding of PsFL-Oct-1 to pCOLADuet-1-based plasmids is likely due to the presence of the Oct-1 specific DNA binding sequence (5′-ATGCAAAT-3′) in a *lacI* region and non-specific interaction between plasmid DNA and Oct-1 DBD.

### Whole-Cell Biotransformation With IIES for Efficient Production of 2′-FL and 3-FL

**Figure 4:**
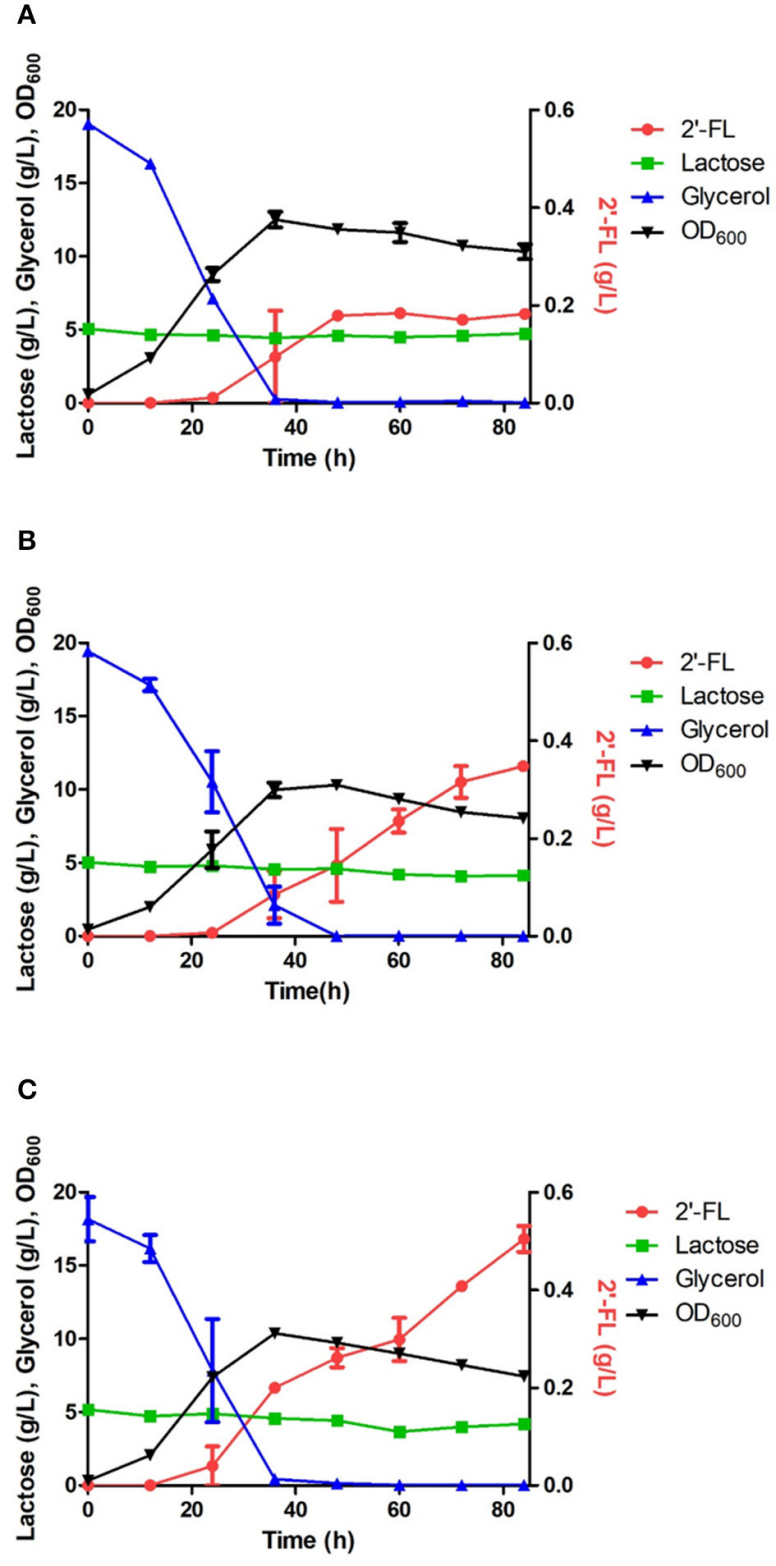
Comparison of 2′-FL production in engineered *E. coli* strains harboring the wild PsFL **(A)**, Oct-1-PsFL **(B)**, and PsFL-Oct-1 **(C)**. When OD_600_ reached 0.4–0.6, IPTG and lactose were added to have a final concentration 0.1 mM and 5 g/L, respectively. Results are the mean of triplicate experiments and error bars indicate s.d.

An engineered *E. coli* capable of producing 2′-FL and 3-FL was constructed by amplifying fluxes in the GDP-l-fucose biosynthetic pathway and by introducing heterologous FTs (Chin et al., 2015; Yu et al., 2018). Specifically, the ΔL M15 BCGW strain was constructed by overexpressing the genes (*manB, manC, gmd*, and *wcaG*) coding for the enzymes in the biosynthetic GDP-l-fucose pathway along with attenuation of the *lac* operon which retains 3% of specific β-galactosidase activity of WT BL21star (DE3). Therefore, the ΔL M15 BCGW strain was chosen as a host strain to produce 2′-FL and 3-FL. Subsequently, the effect of intracellular PsFL immobilization by IIES was investigated with three *E. coli* ΔL M15 BCGW strains containing the WT PsFL (ΔL M15 BCGW-PsFL) and recombinant PsFLs attached with N-terminal (ΔL M15 BCGW-Oct-1-PsFL) and C-terminal (ΔL BCGW-PsFL-Oct-1) Oct-1 DBD in batch fermentations. Although the cell growth pattern and lactose uptake of the three strains were almost identical, we observed different 2′-FL production patterns of *E. coli* strains expressing recombinant PsFLs with Oct-1 DBD. The ΔL M15 BCGW-Oct-1-PsFL strain was able to produce 0.36 g/L of 2′-FL with a yield of 0.33 g 2′-FL/g lactose, showing 2.0-fold (*P*_*value*_ = 0.008) increase of the titer compared to that obtained with the control strain expressing the WT PsFL (Figures 4A,B). Meanwhile, expression of immobilized PsFL with C-terminal Oct-1 DBD (PsFL-Oct-1) resulted in 2.7 (*P*_*value*_ = 0.012) fold higher 2′-FL titer than that of the control strain (Figure 4C). It is worthy to note that the difference in maximum 2′-FL concentrations reached by *E. coli* ΔL M15 BCGW strain expressing the PsFL fusion protein with and without 3 OBS was not statistically significant (Supplementary Figure 5). Collectively from these observations, we concluded that soluble and stable expression of recombinant PsFL by IIES could enable *E. coli* ΔL M15 BCGW background strain to better produce 2′-FL than the control strain.

**Figure 5:**
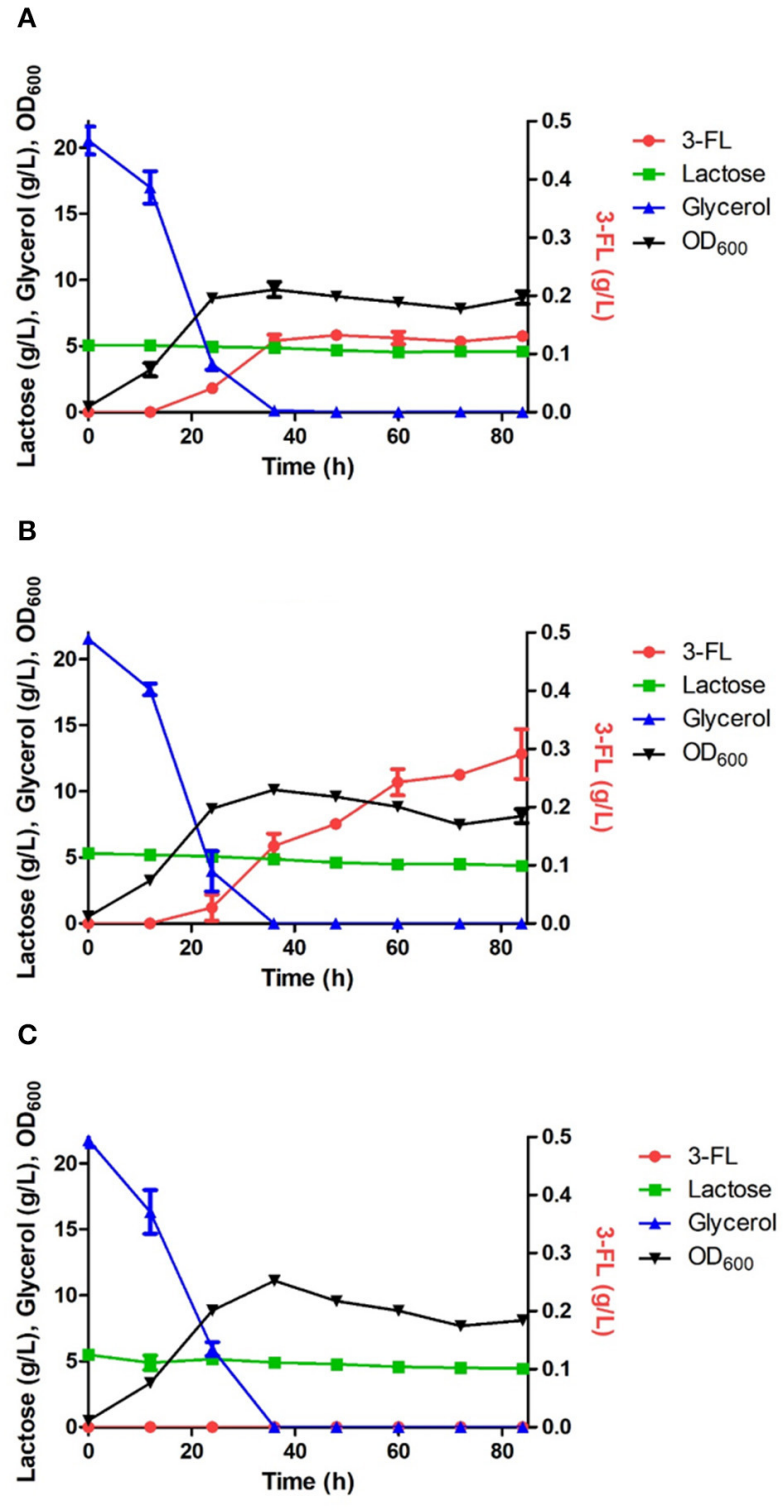
Comparison of 3-FL production in the engineered *E. coli* strains harboring the wild HpFL **(A)**, Oct-1-HpFL **(B)**, and HpFL-Oct-1 **(C)**. When OD_600_ reached 0.4–0.6, IPTG and lactose were added to a final concentration 0.1 mM and 5 g/L, respectively. Results are the mean of triplicate experiments, and the error bars indicate s.d.

The positive effects of enzyme immobilization by plasmid display on biotransformation efficiency was also confirmed for 1,3-FT from *H. pylori* (HpFL). In a batch fermentation with the ΔL M15 BCGW-Oct-1-HpFL strain expressing recombinant HpFL attached with N-terminal Oct-1 DBD, 0.3 g/L of 3-FL was produced, corresponding to 2.3 times (*P*_*value*_ = 0.060) higher than that obtained with the control strain expressing the WT HpFL (ΔL M15 BCGW-HpFL) (Figure 5). However, the engineered strain (ΔL M15 BCGW-HpFL-Oct-1) expressing recombinant HpFL attached with C-terminal Oct-1 DBD could not produce 3-FL in the culture medium (Figure 5C). It is likely that Oct-1 DBD fusion to right after the heptad repeat inhibited dimerization of HpFL molecules which plays an important role in enzyme activity of HpFL. These results indicate that Oc-1 fusion may also increase the stability of oligomeric structure of enzymes when located appropriately.

## Discussion

Whole-cell biotransformation is a viable alternative for production of proteins and chemicals but has some intrinsic disadvantages including rapid degradation or aggregation of unstable enzymes, such as FTs. To circumvent this problem, previous studies have reported *in vivo* immobilization of enzymes using inclusion bodies as a matrix (Steinmann et al., 2010; Han et al., 2017). Multiple heterologous enzymes were successfully immobilized on the surface of inclusion bodies in *E. coli* while the enzyme activities were maintained (Han et al., 2017). However, this system has two inherent possible risks. First, large production of inclusion bodies might cause burden to the cellular protein synthesis machinery, leading to low yield of soluble expression of target enzymes. Second, the formation of denatured and aggregated target enzymes could be accelerated because the inclusion body-forming protein might interact with the newly synthesized enzymes of interest.

Consequently, we sought to develop a system in which the target enzyme is synthesized and simultaneously bound to plasmid DNA that is stably maintained in *E. coli*. Several examples which link proteins to plasmid encoding target genes in *E. coli* cytoplasm have been described recently. DNA binding proteins have been used for plasmid displays include human NF-κB p50 (Speight et al., 2001), yeast GAL4 DNA-binding domain (Choi et al., 2005), zinc finger protein (Rogers et al., 2008), and human Oct-1 DBD (Park et al., 2013). Among these DNA binding proteins, we used the Oct-1 DBD as a DNA binding module due to the following beneficial characteristics: (1) its small size, (2) its short binding site, (3) no requirement of Zn^2+^ for its binding activity, (4) high binding affinity (K_D_ = 9 × 10^−11^ M) via cooperative binding, and (5) its high solubility under optimal *E. coli* growth conditions. Therefore, the highly soluble Oct-1 DBD could be used not only for *in vivo* enzyme immobilization by plasmid display but also for soluble expression of target enzymes.

Stoichiometric mismatch between the number of BS in plasmids, whose copy number is 30~50/cell, and the number of expressed fusion proteins could lead to limited immobilization of target proteins. In this study, however, substantial amounts of PsFL-Oct-1 were bound to pCOLADuet-1-based plasmids regardless of the presence of 3 OBS. While canonical target DNA binding sequences (5′-ATGCAAAT-3′) is the strongest Oct-1 DBD binding site, the degenerate octamer motif (5′-ATGCAAAT-3′) has ~3-fold lower affinity (Verrijzer et al., 1992). This result is consistent with a previous study reporting that most DNA binding proteins capable of binding to specific DNA sequence also share a considerable affinity for non-specific DNA sequences (Kalodimos et al., 2004). The non-specific binding of Oct-1 DBD to plasmid DNA is one of the beneficial characteristics of IIES using Oct-1 fusion protein, as many commercially available plasmids could be used for plasmid display without introduction of 3 OBS. We also note that some Oct-1 fusion proteins might be immobilized on *E. coli* genomic DNA because *E. coli* K-12 strain contains 84 Oct-1 specific DNA binding sequences. Additional experiments are underway to elucidate complexes between Oct-1 fusion protein and *E. coli* genomic DNA.

The utility of the IIES via plasmid display was examined in whole-cell biotransformation processes for production of 2′-FL and 3-FL by assuming that immobilization of the PsFL and HpFL on plasmid plays an important role in thermal stability of enzymes. Indeed, functional expression and stability of the enzymes in *E. coli* under 25°C was significantly improved via IIES using plasmids as a matrix. A previous study reported that Oct-1 DBD is highly soluble and stable in *E. coli* (Park et al., 2013). Therefore, we speculate that attachment of Oct-1 DBD led to stabilization of the enzymes not only because the enzymes are immobilized to plasmid DNA but also because the enzymes are stabilized by fusion of highly stable Oct-1 DBD. While Oct-1 DBD attached at either the N-terminal or C-terminal end of PsFL could enhance the biotransformation activity of the engineered *E. coli*, production of 3-FL was not detected when Oct-1 DBD tag was attached to the C-terminal end of HpFL. Even though the mechanism remains to be investigated, we hypothesize that one of the reasons might be due to the presence of unique heptad repeats in C-terminal region of HpFL. These heptad repeats function as a stem, leading to dimerization of HpFL, which is crucial for stability of the enzyme (Yu et al., 2018). Therefore, it was assumed that the Oct-1 DBD got attached to the C-terminal end of HpFL interfered with heptad repeat-mediated dimerization of HpFL.

In conclusion, *in vivo* immobilization of enzymes via plasmid display allowed soluble and stable expression of the PsFL and HpFL in *E. coli*. As a result, the whole-cell biotransformation efficiency was significantly enhanced by IIES using Oct-1 fusion protein. Our approach could be applicable to other biotransformation processes involving unstable enzymes, whose solubility and stability should be improved for economical production.

## Data Availability Statement

The raw data supporting the conclusions of this article will be made available by the authors, without undue reservation, to any qualified researcher.

## Author Contributions

YP, JS, and D-HK conceived the idea. YP, JS, HK, YJ, HO, YK, JY, JH, MP, and CB designed and performed all experiments. YP, JS, MP, CB, KJ, S-KK, and D-HK analyzed the data. KJ and S-KK provided helpful comments and discussion. D-HK supervised the study. YP, S-KK, and D-HK wrote the manuscript.

### Conflict of Interest

The authors declare that the research was conducted in the absence of any commercial or financial relationships that could be construed as a potential conflict of interest.
