# Supplementary material for: Plasmid Display for Stabilization of Enzymes Inside the Cell to Improve Whole-Cell Biotransformation Efficiency
Source: Front Bioeng Biotechnol. 2020 Jan 10;7:444. doi: 10.3389/fbioe.2019.00444 (PMC6967079; doi:10.3389/fbioe.2019.00444)
Supplement: Supplementary file 1 [file Data_Sheet_1.docx]

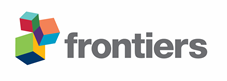
Supplementary Material

**Supplementary Tables**

Table S1. List of primers used.

| Name | Sequence (5’ → 3’) |
| --- | --- |
| pCOLA-Oct-1 V FW | GAGCTCGGCGCGCCTGCAG |
| pCOLA-Oct-1 V BW | GTGGTGATGATGGTGATGGCTGCTGC |
| pCOLA-Oct-1 I FW | CACCATCATCACCACATGGAGGAGCCCAGTGACCTTGA |
| pCOLA-Oct-1 I BW | AGGCGCGCCGAGCTCTGGGTTGATTCTTTTTTCTTTCTGGCGGC |
| pCOLA-BS_FW | TTTTATGGTACCTGTATGCAAATAAGGTACCAAGAAAC |
| pCOLA-BS_BW | GTTTCTTGGTACCTTATTTGCATACAGGTACCATAAAA |
| pCOLA-Oct-1PsFL V BW | TGGGTTGATTCTTTTTTCTTTCTGGCGGCGATTAC |
| pCOLA-Oct-1PsFL I FW | AAAAGAATCAACCCAGGTGGAGGCGGTAGCGGAGGCGGAGGGTCGATGATATTTGTAACCGGATATGGCCAG |
| pCOLA-Oct-1PsFL I BW | AGGCGCGCCGAGCTCTTAAATAATGTGTCGAAACAGATTCTCGAAC |
| pCOLA-PsFLOct-1 V FW | ATGGAGGAGCCCAGTGACCTTGAGGA |
| pCOLA-PsFLOct-1 I FW | CACCATCATCACCACATGATATTTGTAACCGGATATGGCCAG |
| pCOLA-PsFLOct-1 I BW | ACTGGGCTCCTCCATCGACCCTCCGCCTCCGCTACCGCCTCCACCAATAATGTGTCGAAACAGATTCTCGAACTT |
| pCOLADuet V FW | TAAAGAAACCGCTGCTGCGAAATTTGAA |
| pCOLADuet V BW | ATGTATATCTCCTTCTTATACTTAACTAATATACTAAGA |
| Oct-1-PsFL I FW | GAAGGAGATATACATATGGGCAGCAGCCATCACCAT |
| Oct-1-PsFL I BW | TTAAATAATGTGTCGAAACAGATTCTCGAACTTG |
| PsFL-Oct-1 I FW | GAAGGAGATATACATATGGGCAGCAGCCATCACCAT |
| PsFL-Oct-1 I BW | GCAGCGGTTTCTTTATGGGTTGATTCTTTTTTCTTTCTGGCG |
| pCOLADuet-Oct-1-POI V FW | TAAAGAAACCGCTGCTGCGAAATTTGAA |
| pCOLADuet-Oct-1-POI V BW | CGACCCTCCGCCTCCGC |
| pCOLADuet-POI-Oct-1 V FW | GGTGGAGGCGGTAGCGG |
| pCOLADuet-POI-Oct-1 V BW | ATGTATATCTCCTTCTTATACTTAACTAATATACTAAGA |
| Oct-1-HpFL I FW | GGAGGCGGAGGGTCGATGTTCCAGCCCCTTCTCGATG |
| Oct-1-HpFL I BW | GCAGCGGTTTCTTTAGGTATTCTGGCTCAATTCCAGCAG |
| HpFL-Oct-1 I FW | GAAGGAGATATACATATGTTCCAGCCCCTTCTCGATG |
| HpFL-Oct-1 I BW | GCTACCGCCTCCACCGGTATTCTGGCTCAATTCCAGCAG |
| Check FW | AATAATACTGTTGATGGGTGTCTGGTCA |
| Check BW | GAAATCCCTGGTAGTCTCTAAAATACCAGCCTTTGG |
| Gel shift FW | GGGCCTCTAAACGGGTCTTGA |
| Gel shift BW | CAAGGGGTTATGCTAGTTATTGCTCAG |

## Supplementary Figures

**
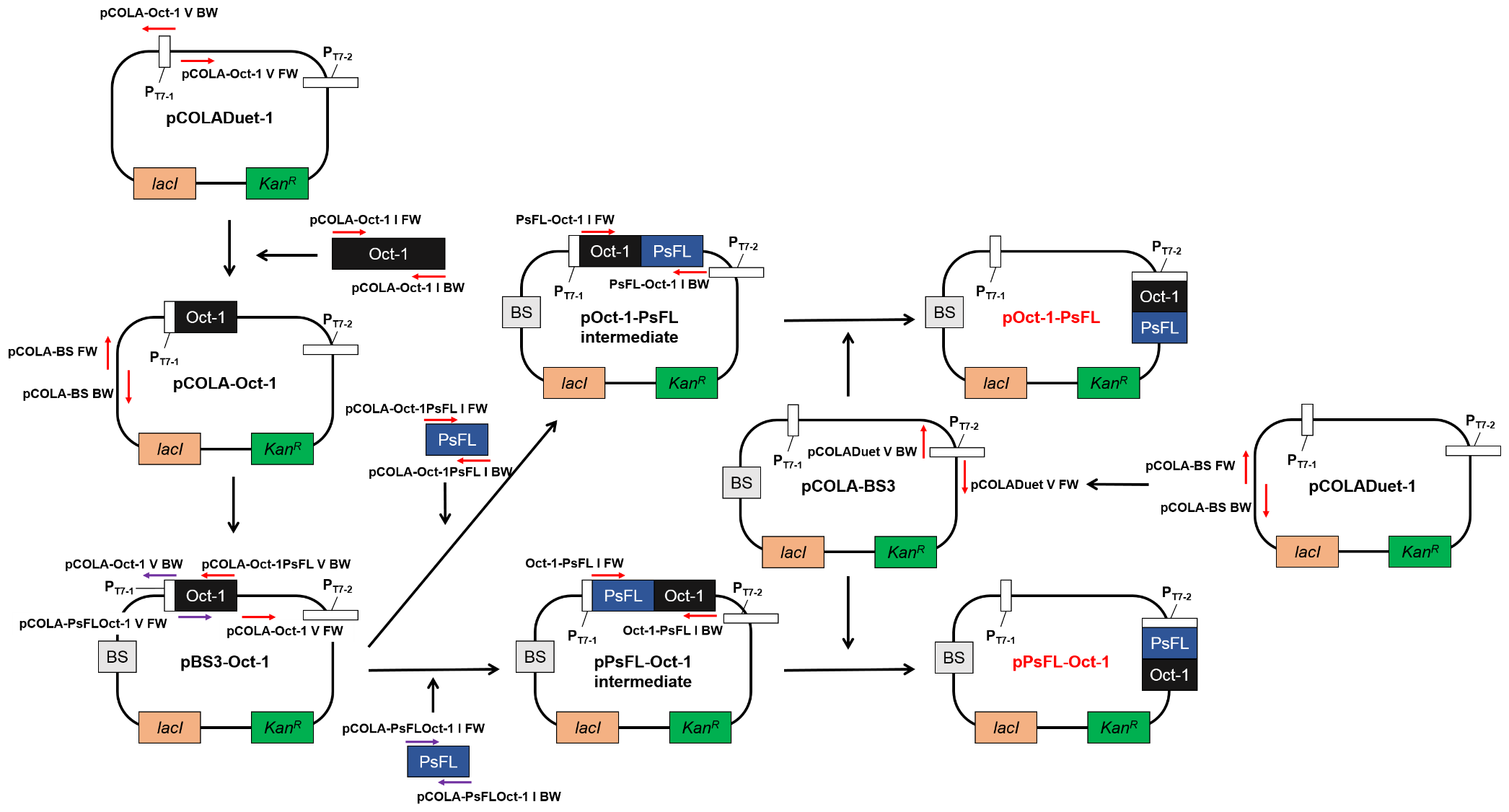
**

**Supplementary Figure 1.** **Schematic of the construction of plasmids pOct-1-PsFL and pPsFL-Oct-1**

**
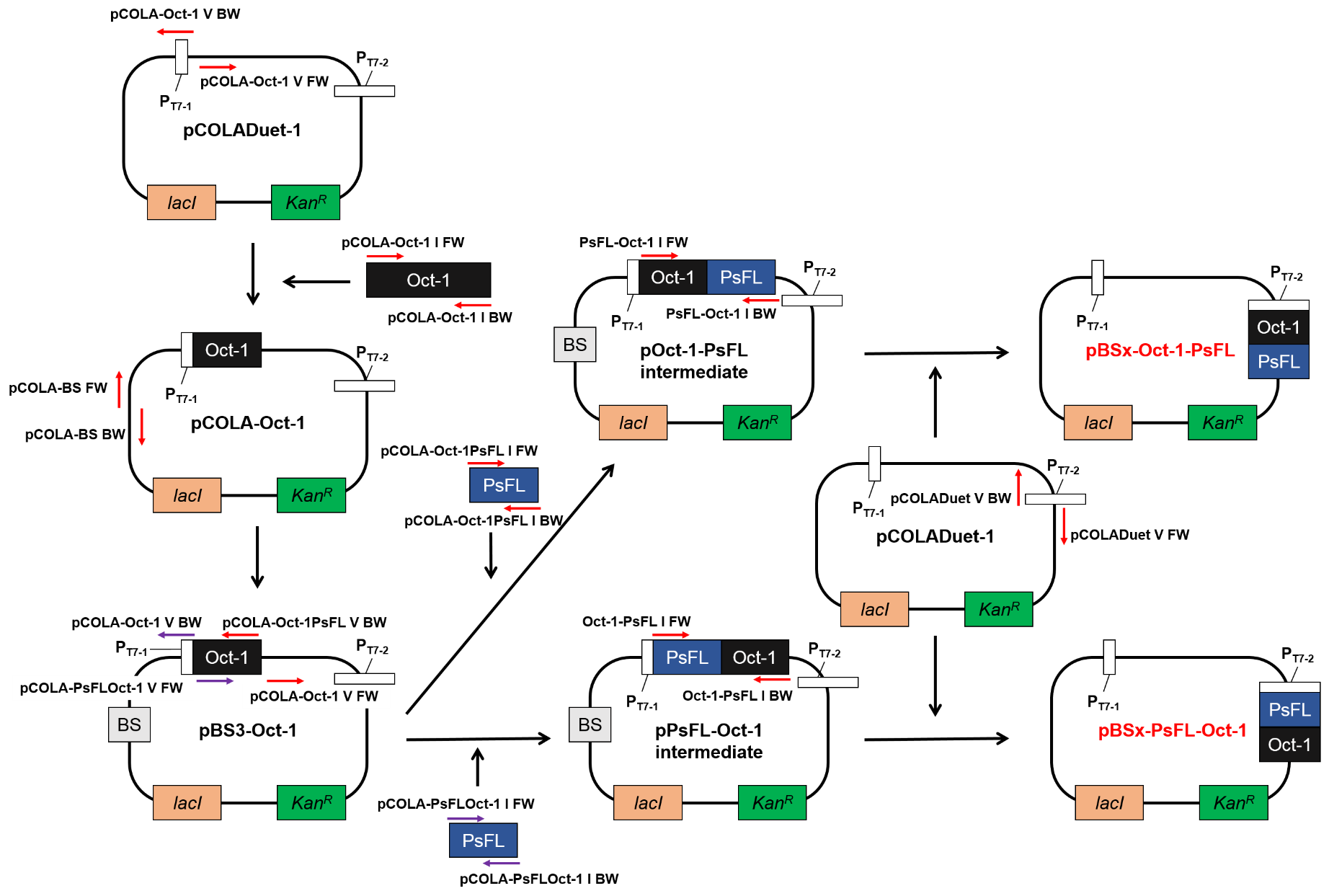
**

**Supplementary Figure 2.** **Schematic of the construction of plasmids pBSx-Oct-1-PsFL and pBSx-PsFL-Oct-1**

**
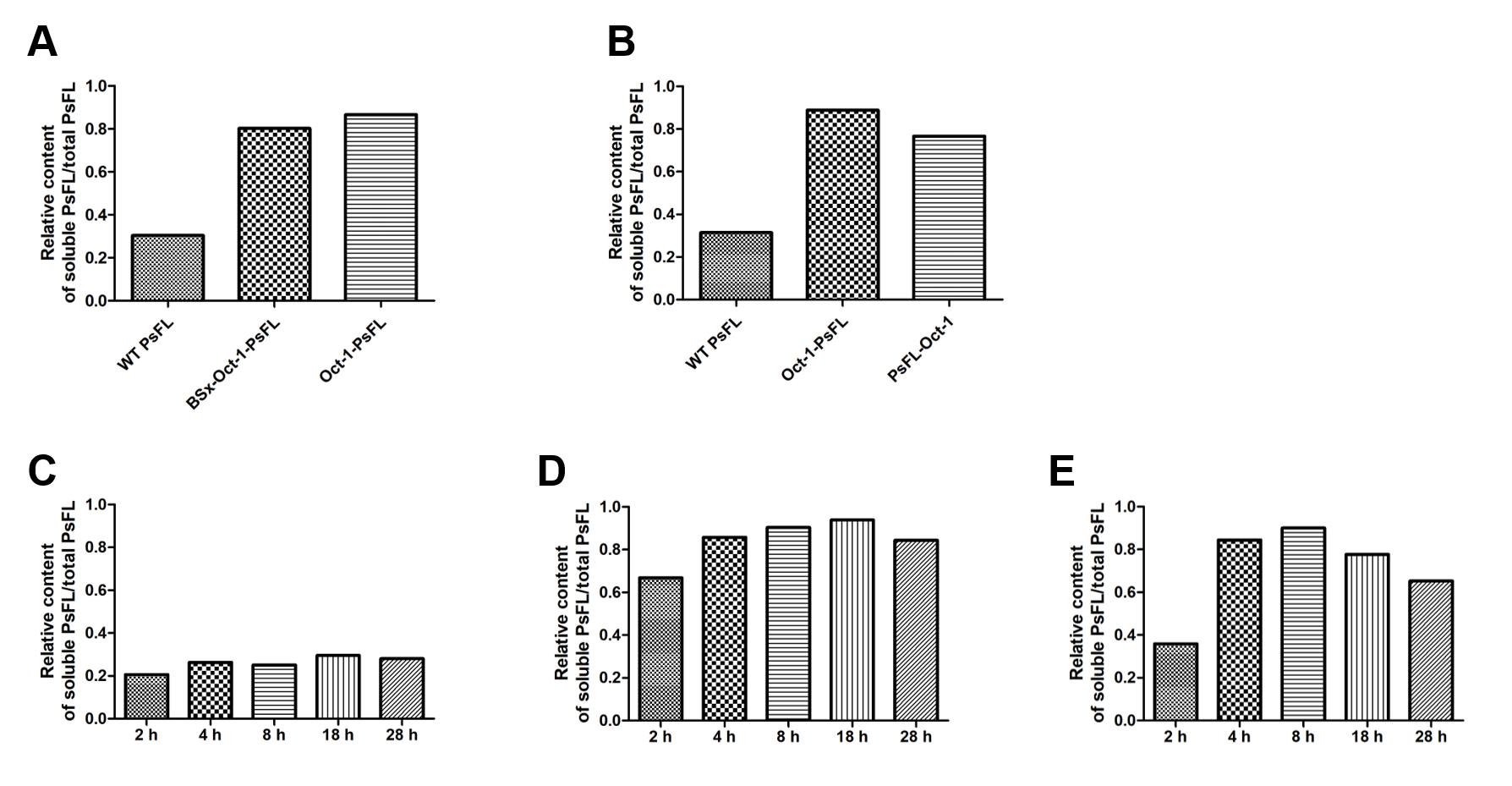
**

**Supplementary Figure 3.** **Relative content of soluble PsFL/total PsFL calculated by densitometric analysis of PsFL bands in the picture of SDS-PAGE presented in Fig. 2.**

**
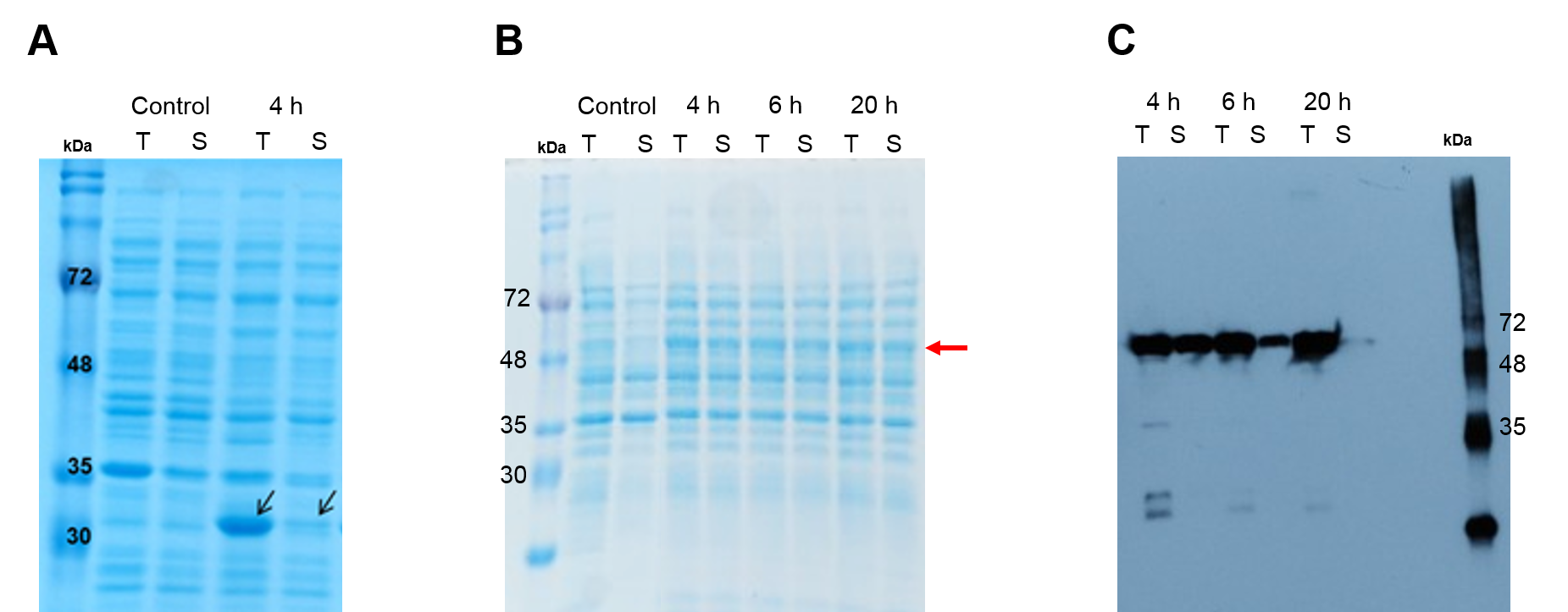
**

**Supplementary Figure 4.** **Effects of Oct-1 DBD fusion on thermal stability of PsFL.** (A and B) SDS-PAGE analysis of WT PsFL (A) and Oct-1-PsFL (B). *E. coli* BL21(DE3) strains expressing recombinant PsFLs were grown at 25°C for 4 h after 0.1 mM IPTG induction. After collecting cells and removing IPTG, cell lysates were prepared from *E. coli* cells re-inoculated into fresh LB medium and cultivated for another 4, 6, and 20 h. The arrow points the protein band of recombinant PsFLs. (C) Western blot analysis of Oct-1-PsFL. The same samples prepared in (B) were analyzed.


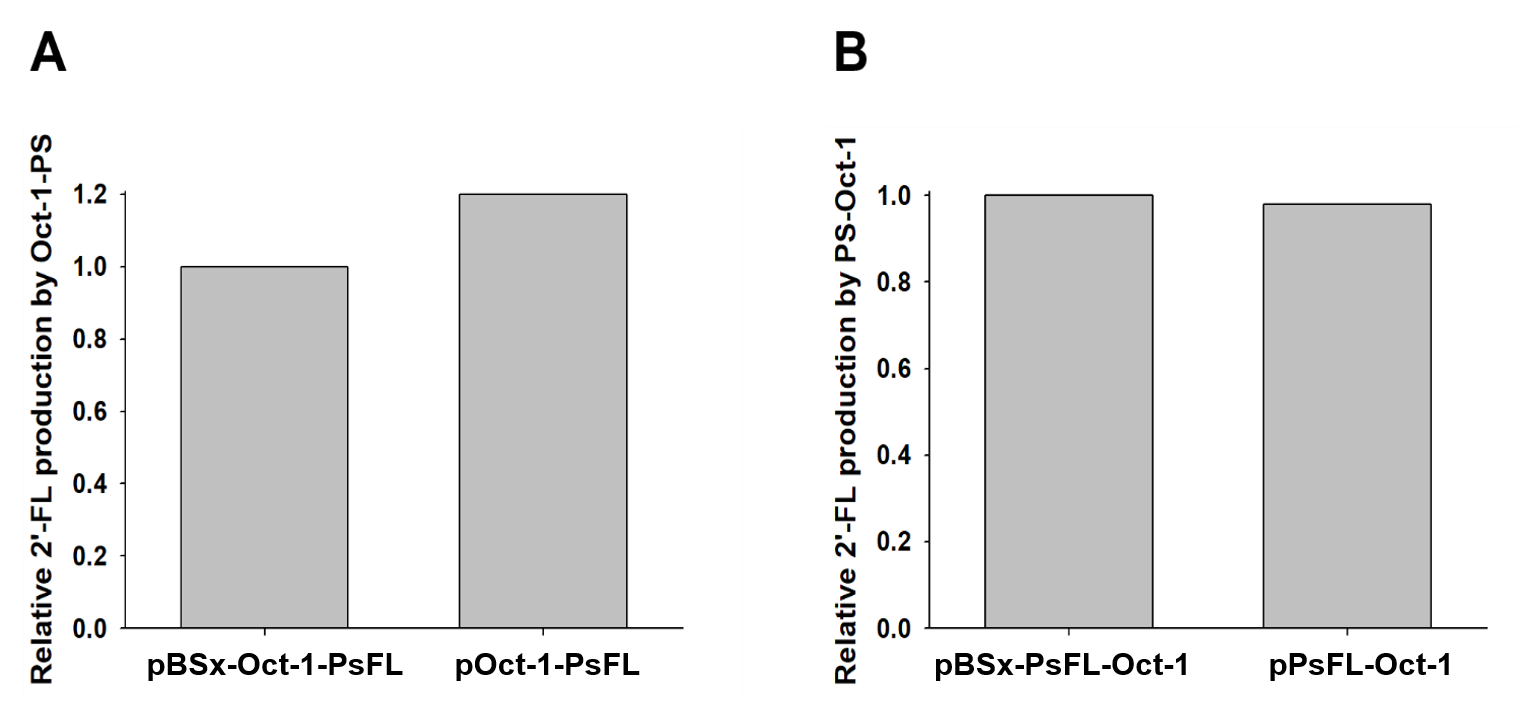


**Supplementary Figure 5.** **Relative 2'-FL production by Oct-1-PsFL (A) and PsFL-Oct-1 (B).**
